# Analysis of neuroretinal rim distribution and vascular pattern in eyes with presumed large physiological cupping: a comparative study

**DOI:** 10.1186/1471-2415-14-72

**Published:** 2014-05-27

**Authors:** Flavio SS Lopes, Syril Dorairaj, Daniela LM Junqueira, Rafael L Furlanetto, Luis Gustavo Biteli, Tiago Santos Prata

**Affiliations:** 1 Department of Ophthalmology, Federal University of São Paulo, Rua Sena Madureira, 1500 - Vila Mariana, São Paulo, SP 04021-001, Brazil; 2 Glaucoma Unit, Hospital Medicina dos Olhos, Rua Salém Bechara, 297 - Centro, Osasco, SP 06018-180, Brazil; 3 Department of Ophthalmology, Mayo Clinic, 4500 San Pablo Road, Jacksonville, FL, USA

**Keywords:** Glaucoma suspect, Intraocular pressure, Optic disc cup, Disc size

## Abstract

**Background:**

To investigate possible differences in neuroretinal rim distribution, vascular pattern, and peripapillary region appearance between eyes with presumed large physiological optic disc cupping (pLPC) and eyes with minimal optic disc excavation.

**Methods:**

We prospectively enrolled consecutive subjects with pLPC and individuals with minimal excavation (optic disc excavation within normal limits; control group). All eyes had normal visual fields and untreated intraocular pressure (IOP) <21 mmHg. Eyes with pLPC required vertical cup-to-disc ratio (VCDR) ≥0.6 and ≥30 months of follow-up with no evidence of glaucomatous neuropathy. For controls, VCDR was limited to ≤0.5. We compared ocular signs and characteristics related to the neuroretinal rim distribution, vascular pattern, peripapillary region appearance and disc size between groups. Whenever both eyes were eligible, one was randomly selected for analysis.

**Results:**

A total of 74 patients (mean age, 45.6 ± 14.9 years) with pLPC and 45 controls (mean age, 44.8 ± 11.6 years) were enrolled (p = 0.76). Median disc size and VCDR was significantly larger in eyes with pLPC compared to controls (p < 0.01). The proportion of eyes with violation of the ISNT rule, laminar dot sign, nasal shifting of the central vessels, nasal excavation and baring of circumlinear vessel was significantly greater in the eyes with pLPC compared to controls (p < 0.01). There were no significant differences regarding the proportions of eyes with peripapillary atrophy between groups (p < 0.09). Finally, disc size was significantly associated with VCDR (r^2^ = 0.47, p < 0.01), with an increase of 0.21 in VCDR for each 1 mm^2^ in disc area.

**Conclusion:**

Compared to normal controls, eyes with pLPC may present a higher proportion of optic nerve head findings frequently observed in glaucomatous eyes. This seems to be explained in part by the larger discs found in these eyes. We believe care should be taken while classifying them as glaucomatous or not based solely on these characteristics.

## Background

Glaucoma is usually diagnosed by observation of structural changes in the optic nerve head (ONH) and functional loss as determined by standard automated perimetry [1]. Although cup-to-disc ratio (CDR) has long been used in the evaluation of the glaucoma suspect, the wide range of CDR values in the normal population (from 0.00 to 0.87) and the high interindividual variability of optic disc area (0.8 and 6.0 mm^2^) limits its use [2-5]. It is generally recognized that a large CDR might be physiologic if the optic disc is large [6], while even a small CDR might mean glaucomatous optic neuropathy in the presence of a small disc [7].

Among all glaucoma suspects, eyes with ONH features suspicious or suggestive of early glaucoma are probably those that offer the greatest challenge for clinicians. In contrast with the robust longitudinal data published on ocular hypertensive glaucoma suspects [8-12], there are few specific management guidelines for patients with suspicious ONH appearance. In this scenario, at the time of diagnosis, it is not an easy task to determine whether a patient has glaucoma or just a large physiological optic disc cup.

Currently, clinicians often rely on well-established patterns and signs of normality to evaluate these eyes with suspicious large disc cups. However, all these normal characteristics are derived from the comparison between glaucomatous patients and individuals with definitely healthy eyes (normal cupped discs). It is not known whether eyes with presumed large physiological disc cups (pLPC; such as those found in eyes with large discs) would have the same morphological characteristics as normal small-cupped eyes. The purpose of the present study was to investigate possible differences in neuroretinal rim distribution, vascular pattern, and peripapillary region appearance between eyes with pLPC and eyes with minimal excavation (optic disc excavation within normal limits; control group).

## Methods

This prospective protocol adhered to the tenets of the declaration of Helsinki and was approved by the institutional review board of the Federal University of São Paulo. In addition, written informed consent was obtained from all participants.

### Participants

In this observational case–control study, we prospectively enrolled consecutive patients with pLPC and subjects with minimal optic disc excavation (control group). All participants underwent a comprehensive ophthalmological evaluation, including best-corrected visual acuity, slit-lamp biomicroscopy, intraocular pressure (IOP) measurement, gonioscopy, dilated fundoscopy, visual field (VF) testing, optic disc stereophotographs, color and red-free fundus imaging, and spectral-domain optical coherence tomography (SD-OCT; RTVue-100; Optovue, Inc., Fremont, CA; software version A4).

Eyes with pLPC were defined as those with vertical cup-to-disc ratio (VCDR) ≥0.6 (based on color stereophotography evaluation), untreated IOP ≤20 mmHg, normal VF tests (Humphrey SITA - Standard 24–2, Carl Zeiss Meditec, Dublin, CA), and absence of disc notching, disc hemorrhage, or localized retinal nerve fiber layer defect. Controls were recruited based on the same criteria, except for VCDR, which was limited to ≤0.5. Based on the ISGEO classification, in most studies the VCDR cut-off value for classifying an optic disc as glaucomatous was usually determined as ≥ 0.7 (based on the 97.5 percentile of the CDR distribution for the studied population [13,14]). In the present study, our goal was to separate participants in healthy and suspect eyes (based on disc appearance), not glaucomatous. Therefore, we adopted a less strict cut-off value (≥0.6), which we considered more clinically relevant on daily practice, as many eyes with a CDR of 0.6 would be probably classified as suspects on a clinical scenario. Finally, all included patients had to have a minimum follow-up of 30 months, with no changes in optic disc or VF parameters. We adopted the same structural and functional criteria used for determining glaucoma conversion previously described in the Ocular Hypertension Treatment Study [15]. During follow-up, all participants (cases and controls) were examined every 6 months. In every visit, each patient underwent IOP measurement, dilated fundoscopy, VF testing, optic disc stereophotographs, color and red-free fundus imaging. Therefore, all eyes had disc photos and VFs at the beginning and at the end of the follow-up period. Disc size (through SD-OCT) was assessed only at baseline.

### Data collection

Demographic and ocular characteristics were collected and compared between groups. For the analyses and comparisons of the characteristics and signs related to the neuroretinal rim distribution (violation of the ISNT rule [5], nasal excavation, and laminar dot sign), peripapillary region (beta zone peripapillary atrophy) and vascular pattern (nasal shifting of the central vessels and baring of the circumlinear vessel) between groups, two experienced examiners (FSL and TSP; both glaucoma specialists) assessed all digital photographs masked to patient’s clinical data. In cases of disagreement, the opinion of a third examiner (RF; glaucoma specialist) was used to adjudicate. The classification of each patient was based on a standardized photographic guide (based on images from the book “The optic nerve in glaucoma” [16]) containing examples of each different sign and characteristic that was being investigated. Objective disc size measurements were obtained from SD-OCT results [17]. Whenever both eyes were eligible, one was randomly selected for analysis.

### Statistical analysis

Descriptive analysis was used to present demographic and clinical data. D’Agostino–Pearson’s test was performed to determine whether the data had a normal distribution. Independent samples *T*-test was used to compare continuous normally distributed variables between groups, while the Mann–Whitney test was used to compare those non-normally distributed. Categorical data were compared using chi-square and Fisher’s exact tests. Regression analysis was used to evaluate the association between disc size and VCDR.

The violation of the ISNT rule was chosen as the clinical parameter for sample size calculation. Our initial sample size calculation was based on a hypothesized difference of 30% (between the frequencies in each group) as clinically significant and an α-error of 0.05. Considering two groups with the same number of patients, we would need to include a total of 90 participants (45 in each group) to reach a statistical power of 80% (beta or type II error of 0.20). However, after enrollment, we ended up with an uneven number of participants in each group (74 patients and 45 controls). Therefore, we conducted another sample size calculation (post-hoc) based on this uneven sample size distribution among the two groups. Considering this sample size ratio of 1.6 (cases/controls), we detected that it would be necessary a total of 98 participants (a minimum of 60 cases and 38 controls) in order to maintain a statistical power of 80%. Computerized analysis was performed using MedCalc software (MedCalc Inc., Mariakerke, Belgium).

## Results and discussion

A total of 119 participants (74 patients with pLPC and 45 controls) were enrolled. Table 1 provides clinical and demographic characteristics of included patients. Age and gender distribution were similar between the two groups (p ≥ 0.76). Median disc size and VCDR were significantly larger in eyes with pLPC compared to controls (p < 0.01). The proportions of eyes with violation of the ISNT rule, laminar dot sign, nasal shifting of the central vessels, nasal excavation, and baring of the circumlinear vessel were significantly greater in eyes with pLPC compared to controls (p < 0.01). There was no significant difference regarding the proportions of eyes with peripapillary atrophy between groups (p < 0.09). Table 2 provides a more detailed comparison between groups. Finally, disc size was significantly associated with VCDR (r^2^ = 0.47, p < 0.01), with an increase of 0.21 in VCDR for each 1 mm^2^ in disc area.

**Table 1:** Demographic and ocular characteristics of patients with presumed large physiological cupping (LPC) and controls

**Parameters**	**Presumed LPC (n = 74)**	**Controls (n = 45)**	**P value**
Age (years)*	45.6 ± 14.9	44.8 ± 11.6	0.76
Sex (%; M/F)	38/62	35/65	0.85
Race (%; W/B/O)	79/15/6	71/20/9	0.66
Disc size^†^ (mm^2^)	2.50 (2.3 to 2.8)	1.86 (1.5 to 2.1)	<0.01
Average RNFL thickness* (μm)	107.03 ± 8.51	109.34 ± 7.16	0.18
Vertical cup-to-disc ratio^†^ (mm^2^)	0.7 (0.6 to 0.7)	0.4 (0.3 to 0.5)	<0.01

LPC, large physiological cupping; M, male; F, female; W, White; B, Black; O, Others; RNFL, retinal nerve fiber layer.

*Data are given as mean ± standard deviation.

†Non-normally distributed variables represented by median (first quartile, third quartile).

**Table 2:** Ocular characteristics of patients with presumed large physiological cupping and controls

**Characteristics**	**Presumed LPC %**	**Controls %**	**P value**
Violation of the ISNT rule	37.8	8.9	<0.01
Peripapillary atrophy	54.1	37.8	0.09
Laminar dot sign	45.9	15.6	<0.01
Nasal shifting of the central vessels	47.3	6.7	<0.01
Nasal excavation	28.3	2.2	<0.01
Baring of the circumlinear vessel	22.9	2.2	<0.01

Although a common scenario on a clinical setting, there is scant information in the literature or guidelines about how we should manage patients with suspicious appearance of the optic disc and IOP within the normal range. Most of the available guidelines refer to glaucoma suspects with ocular hypertension. For these patients, there are well-established risk factors. Looking carefully at the AAO Glaucoma Suspect PPP (Preferred Practice Pattern®”), although most of the data refer to glaucoma suspects defined by high IOP values (and not to those defined by a suspicious disc appearance), the main recommendations state that glaucoma suspects need periodic monitoring of IOP values, visual field status, optic disc appearance, and retinal nerve fiber layer status, in order to determine if damage has occurred. Therefore, unless a patient presents with anatomical (eg, localized peripapillary retinal nerve fiber layer or neuroretinal rim defects) or functional findings strongly suggestive of glaucomatous neuropathy, he or she could be followed as a suspect indefinitely.

In clinical practice, the differentiation between glaucomatous and non-glaucomatous cupping can be difficult. This situation seems to occur more often in cases of large discs. While evaluating patients with suspicious large cupped discs, we usually apply the same diagnostic criteria and search for the same anatomical patterns and characteristics associated with the presence (or absence) of glaucoma, independent of the disc size. Therefore, we assume that eyes with large physiological disc cups would have the same morphological characteristics as normal small-cupped eyes. In the present study, comparing eyes with pLPC (followed for more than two years) and eyes with minimal excavation (optic disc excavation within normal limits), we found that many of the signs usually suggestive of glaucoma are also frequently observed in these large cupped, but non-glaucomatous eyes. This seems to be explained in part by the larger discs found in these eyes. The authors are unaware of any previously published study with a similar purpose and design.

The term pseudoglaucomatous physiologic large cup was introduced by Jonas et al. [6]. Some of the described morphologic characteristics of these cases were an abnormally large optic disc area, large CDR, normal neuroretinal rim area and configuration, normal form of alpha zone, no beta zone and normal parapapillary retinal nerve fiber layer. When considering disc size and peripapillary region findings, we believe our results corroborate most of these previous described characteristics [6,18], as we found larger discs and a similar proportions of eyes with peripapillary atrophy between groups. Regarding neuroretinal distribution characteristics, we found a higher percentage of eyes with violation of the ISNT rule in the pLPC group (37.8). This is almost twice as the percentage reported in healthy eyes (21%) by Harizman et al. [19]. It is important to highlight that healthy eyes from the above cited study had to have a VCDR ≤ 0.6 and an asymmetry between eyes <0.2. In another interesting paper investigating the violation of the ISNT rule in glaucomatous and healthy eyes [based on HRT (Heidelberg Retinal Tomograph) measurements], Chan et al. [20] found that disc area had a significant impact on the ISNT rule diagnostic performance. Although they reported high sensitivity and specificity values for the ISNT rule and its variants, they found a lower specificity in cases of large disc areas. We believe our findings corroborate the above-mentioned results. Therefore, although the ISNT rule has been largely applied to differentiate glaucomatous from normal cupped healthy eyes, it doesn’t seem to be a good diagnostic parameter to use while dealing with eyes with large cups associated with large discs. Finally, we found that other ONH and vascular characteristics (such as baring of the circumlinear vessel and laminar dot sign) were also more frequently documented in eyes with pLPC than in controls. Although not pathognomonic nor strongly specific for glaucoma, all these features (provided in Table 2) were reported in previous studies as being more frequently observed in eyes with glaucoma compared to healthy controls [21-24]. Based on these findings, we believe these features seem to be part of the normal morphological appearance of these eyes, and should not be considered as indicators of the presence of glaucomatous neuropathy.

Optic disc size varies significantly among individuals, ranging between 0.8 and 6.0 mm^2^[4]. It is well-known that a large cup may be physiologic if the optic disc is large [4,6,13]. While clinicians may overdiagnose the disease in eyes with large discs and physiologic cupping, they may miss early glaucoma in eyes with small discs and small cups. We not only found larger discs in the pLPC group compared to controls, but also the disc size explained almost half of the VCDR variation among patients (coefficient of determination of 0.47). Each 1 mm^2^ in disc area seemed to account for a variation of 0.21 in VCDR. We believe that the larger discs found in patients with pLPC are not only related to the larger VCDR values observed in these eyes, but also could explain the differences in neuroretinal rim distribution and vascular pattern documented in comparison to controls (small cupped eyes).

The present study has some specific characteristics and limitations that should be addressed. First, we defined IOP within the normal range (≤20 mmHg) without considering diurnal IOP variation and central corneal thickness influence on applanation tonometry measurements. Second, it is possible that some eyes with pLPC will develop glaucomatous progression over time. By including pLPC eyes with at least 30 months of follow-up without progression, we expect to reduce this occurrence. Third, our results should not be generalized to other populations, as our sample was limited to a single center in Brazil. Fourth, a longitudinal analysis following these pLPC eyes over time is warranted in order to confirm our findings. Finally and most importantly, we may have included a biased set of large physiological cupping eyes, as eyes with additional anatomic features (such as violation of the ISNT rule) may have been more likely to be followed clinically for possible glaucoma than those with large physiological cupping but no other features. Therefore, our results need to be confirmed in a population-based study. These limitations should be considered while interpreting our results.

## Conclusion

Neuroretinal rim distribution and vascular pattern seems to vary significantly between eyes with pLPC and those with minimal optic disc excavation. This seems to be explained in part by the larger discs found in the former group. Since some of the characteristics we documented in eyes with pLPC are also frequently seen in glaucomatous eyes, we believe that care should be taken while classifying them as glaucomatous or not based solely on these characteristics.

## Abbreviations

CDR: Cup-to-disc ratio; IOP: Intraocular pressure; ONH: Optic nerve head; pLPC: Presumed large physiological optic disc cupping; VCDR: Vertical cup-to-disc ratio; VF: Visual field.

## Competing interests

The authors declare no conflict of interest.

## Author’s contributions

FSSL contributed to the collection of data, drafting of the article, generation of figures, and collection of images. SD contributed to the conception and design of the study, analysis and interpretation of the data, and critical revision of the article. DLMJ contributed to the collection of data, generation of figures, and collection of images. RLF contributed to the collection of data, generation of figures, and collection of images. LGB contributed to the conception and design of the study, analysis and interpretation of the data, and critical revision of the article. TSP contributed to the conception and design of the study, analysis and interpretation of the data, drafting of the article, and critical revision of the article. All authors approved the final draft of the article.

## Pre-publication history

The pre-publication history for this paper can be accessed here:

http://www.biomedcentral.com/1471-2415/14/72/prepub
